# Neutrophil Extracellular Traps Promote Pancreatic Cancer Progression via the STING Pathway

**DOI:** 10.1155/grp/4950214

**Published:** 2025-02-26

**Authors:** Mengdi Qu, Chenyu Zhu, Caihong Sun, Shuainan Zhu, Hao Zhang, Changhong Miao, Di Zhou

**Affiliations:** ^1^Department of Anesthesiology, Zhongshan Hospital, Fudan University, Shanghai, China; ^2^Shanghai Key Laboratory of Perioperative Stress and Protection, Shanghai, China; ^3^Department of Anesthesiology, Shanghai Medical College, Fudan University, Shanghai, China; ^4^Department of Anesthesiology, Shanghai Geriatric Medical Center, Shanghai, China

**Keywords:** EMT, IL-8, NETs, pancreatic cancer, STING

## Abstract

**Background:** Pancreatic cancer is very susceptible to metastasis with a high mortality. Neutrophil extracellular traps (NETs) have been reported to be associated with poor prognosis in patients suffering from pancreatic cancer. However, the underlying mechanisms by which NETs facilitate cancer progression remain poorly understood.

**Methods:** The expression of NETs was assessed in pancreatic cancer tissues and plasma samples from patients. Neutrophils were isolated from the blood of individuals diagnosed with pancreatic cancer to evaluate NETs formation. The impact of NETs on the progression of pancreatic cancer cells was investigated, along with a series of experiments aimed at elucidating the interaction mechanisms between neutrophils and cancer cells.

**Results:** Pancreatic cancer samples had higher levels of NETs, and NETs formation was intensified in neutrophils derived from patients. NETs significantly promoted both migration and invasion capabilities in pancreatic cancer cells. Furthermore, the stimulator of interferon genes (STING) signaling pathway was stimulated to produce interleukin-8 (IL-8), which subsequently recruited more neutrophils and mediated further formation of NETs.

**Conclusions:** Our data indicate a NETs–cancer aggressive crosstalk in pancreatic cancer. Specifically, NETs stimulate tumor cells to secrete IL-8, thereby promoting NETosis within the tumor microenvironment. Consequently, NETs may be a key target for pancreatic cancer treatment.

## 1. Introduction

Pancreatic cancer is associated with a poor prognosis due to its aggressiveness and high metastatic potential, ranking as the seventh leading cause of cancer-related mortality [1, 2]. Over half of all patients diagnosed with pancreatic cancer present with metastasis at the time of diagnosis, and the liver is the most common site of distant metastases [3]. Thus, recognizing the mechanisms of metastasis is key to aiding the development of effective therapies.

Epithelial–mesenchymal transition (EMT) is an essential process for tumor metastasis, which is activated by various pathogenic stimuli and enhances the migratory and invasive capabilities of cancer cells [4]. Exploring the mechanisms related to EMT of pancreatic cancer cells will provide us with novel therapeutic targets [5]. Growing evidence has shown that the inflammatory microenvironment would promote carcinogenesis and cancer progression [6, 7]. Furthermore, EMT and invasiveness are most abundant at inflammatory sites, which means that inflammation is necessary for EMT and dissemination [8].

Neutrophils are the most abundant type of leukocytes, being at the forefront of defense against infections, but their role in tumors is still unclear [9, 10]. Neutrophil extracellular traps (NETs) are web-like structures from activated neutrophils, which have been found to be involved in cancer development and metastasis [11–13]. A number of studies highlight NETosis as a potentially promising diagnostic and therapeutic target. Furthermore, NETs were reported to interact reciprocally with pancreatic cancer cells, leading to inflammation-associated metastasis [14]. However, the specific mechanisms by which NETs promote cancer progression have not been fully understood.

In this study, we demonstrated that NETs could aggravate tumor aggressiveness through the induction of EMT. NETs stimulated the stimulator of interferon genes (STING) pathway of cancer cells to produce interleukin-8 (IL-8, also known as CXCL8), which would recruit more neutrophils. Consequently, the crosstalk between tumor cells and neutrophils resulted in a poor prognosis in pancreatic cancer patients.

## 2. Material and Methods

### 2.1. Patients and Specimens

We collected paraffin-embedded tissue sections and plasma samples from patients with pancreatic ductal adenocarcinoma (PDAC) diagnosed at the Zhongshan Hospital, Fudan University. The study protocol was approved by the Ethics Committee of Zhongshan Hospital, Fudan University (protocol license number: B2020-062R2), and all procedures were conducted in accordance with the Declaration of Helsinki. Written informed consent was obtained from all participants before recruitment.

### 2.2. Cell Culture

BxPC-3, MIAPaCa-2, and PANC-1 cell lines were purchased from the American Type Culture Collection (ATCC). BxPC-3 was maintained in Roswell Park Memorial Institute (RPMI) 1640 (Gibco) supplemented with 10% fetal bovine serum (FBS) (Gibco). MIAPaCa-2 and PANC-1 were cultured in Dulbecco's modified Eagle medium (DMEM) (Gibco) containing 10% FBS. All pancreatic cancer cell lines were cultured at 37°C in a humidified atmosphere with 5% CO_2_ and 95% air.

### 2.3. Isolation of Neutrophils and NETs Production

Neutrophils were isolated from human blood according to the kit's instructions (TBD Sciences). Briefly, the blood samples were layered over the neutrophil separation medium and centrifuged at 800 g for 30 min at room temperature. The lower leukocyte layer containing neutrophils was collected, followed by schistocyte and washing with phosphate buffered saline (PBS). The obtained cells were resuspended in different mediums depending on the subsequent experiments. For NETs production, isolated neutrophils were stimulated with 50 nM phorbol 12-myristate 13-acetate (PMA) (MKBio) for 4 h. After removing the supernatant, NETs adhered at the bottom were washed down by adding 2 mL of cell culture medium and were centrifuged to remove cell debris.

### 2.4. Immunohistochemistry (IHC) Assay

Paraffin-embedded tissue sections were deparaffinized, rehydrated, treated with 0.3% hydrogen peroxide, and processed for antigen retrieval with heat induction for approximately 10 min. Primary antibodies to the following proteins were used for IHC staining: myeloperoxidase (MPO) (1:50, R&D Systems, AF3667) and STING (1:100, Cell Signaling Technology, 13647S). Tissues were then incubated with peroxidase-conjugated goat anti-mouse/rabbit IgG secondary antibody. The slides were finally stained with 3,3⁣′-diaminobenzidine and counterstained with hematoxylin.

### 2.5. Immunofluorescence (IF) Assay

After cells were fixed and permeabilized, or paraffin-embedded tissue sections were deparaffinized, rehydrated, and processed for antigen retrieval, 1% bovine serum albumin (BSA) was used to block and then incubated with antibodies against citrullinated histone H3 (CitH3) (1:100, Abcam, ab5103), MPO (1:50, R&D Systems, AF3667), and STING (1:100, Cell Signaling Technology, 13647S) at 4°C for 12 h. Secondary antibodies with Alexa 488 or 594 (1:200, Abcam) were added for 60 min at 37°C. Finally, 4⁣′,6-diamidino-2-phenylindole (DAPI) was used to stain nuclei. The slices were visualized under a confocal microscope.

### 2.6. Cell Proliferation Assay

A Cell Counting Kit-8 (Dojindo Corp.) was used to test the relative cell proliferation rate for 24 h according to the manufacturer's instructions. The absorbance (optical density, OD 450 nm) of each 96-well plate was measured with a Tecan Infinite F50 microplate reader.

### 2.7. Cell Migration and Invasion Assays

Pancreatic cancer cells were seeded into six-well plates and cultured for 12 h to achieve 100% confluence. Cell monolayers were manually wounded by scraping cells with a P200 pipette tip and followed by washing with PBS. Then, the cells were cultured in different mediums containing 1% serum. The wound width was measured at 0 h and 24 h after the scratch.

Cell migration and invasion assays were performed with chambers (Corning) containing 8-*μ*m pores with or without Matrigel (BD Falcon), respectively. Cells were added to the upper chamber with different mediums in the lower chamber. After incubation for 24 h at 37°C, the noninvading cells were wiped off, and cells on the bottom side of the membrane were fixed with methanol and stained with crystal violet. The stained cells were counted under a light microscope. The migration ability of neutrophils was measured with chambers (Millipore) containing 3-*μ*m pores.

### 2.8. Reverse Transcription-Polymerase Chain Reaction (RT-PCR)

Total RNA was extracted with a TRIzol reagent (Sigma-Aldrich). The cDNA was synthesized with a PrimeScript RT reagent kit (RR036A, Takara), and RT-qPCR was performed with a TB Green PCR kit (RR820A, Takara). Quantification of gene expression was normalized to endogenous *β*-actin expression. The primer sequences used were as follows:

Zeb-1 forward: 5⁣′-GATGATGAATGCGAGTCAGATGC-3⁣′

Zeb-1 reverse: 5⁣′-ACAGCAGTGTCTTGTTGTTGT-3⁣′

Snail-2 forward: 5⁣′-CGAACTGGACACACATACAGTG-3⁣′

Snail-2 reverse: 5⁣′-CTGAGGATCTCTGGTTGTGGT-3⁣′

Vimentin forward: 5⁣′-GACGCCATCAACACCGAGTT-3⁣′

Vimentin reverse: 5⁣′-CTTTGTCGTTGGTTAGCTGGT-3⁣′

IL-8 forward: 5⁣′-TTTTGCCAAGGAGTGCTAAAGA-3⁣′

IL-8 reverse: 5⁣′-AACCCTCTGCACCCAGTTTTC-3⁣′

*β*-Actin forward: 5⁣′-CTCGCCTTTGCCGATCC-3⁣′

*β*-Actin reverse: 5⁣′-GAATCCTTCTGACCCATGCC-3⁣′

### 2.9. Western Blot

Cells with or without treatments were harvested and lysed with radio immunoprecipitation assay (RIPA) buffer (Solarbio) containing proteinase inhibitor phenylmethanesulfonyl fluoride (PMSF) (1 mM). Equal amounts of protein were loaded on sodium dodecyl sulfate-polyacrylamide gel electrophoresis and transferred to a polyvinylidene fluoride (PVDF) membrane. The membrane was blocked with 5% milk for 1 h at room temperature and then was incubated with primary antibodies overnight at 4°C, including CitH3 (1:1000, Abcam, ab5103), MPO (1:1000, Abcam, ab208670), E-cadherin (1:1000, Cell Signaling Technology, 3195), N-cadherin (1:1000, Cell Signaling Technology, 13116), vimentin (1:2000, Cell Signaling Technology, 5741), alpha-smooth muscle actin (*α*-SMA) (1:1000, Cell Signaling Technology, 19245), STING (1:1000, Cell Signaling Technology, 13647), phospho-TBK1 (p-TBK1) (1:1000, Cell Signaling Technology, 5483), TBK1 (1:1000, Abcam, ab40676), nuclear factor kappa-light-chain-enhancer of activated B cells (NF-*κ*B) (1:1000, Abmart, TA5006), phospho-MEK (p-MEK) (1:1000, Cell Signaling Technology, 9154), MEK (1:1000, Cell Signaling Technology, 8727), phospho-ERK1/2 (p-ERK1/2) (1:1000, Cell Signaling Technology, 4370), ERK1/2 (1:1000, Cell Signaling Technology, 4695), phospho-Akt (p-Akt) (1:1000, Cell Signaling Technology, 4060), Akt (1:1000, Cell Signaling Technology, 4691), and *β*-actin (1:3000, Cell Signaling Technology, 3700). After being incubated with horseradish peroxidase (HRP)-conjugated secondary antibodies, ECL Western Blotting Substrate (Tanon) was used to detect the protein expression.

### 2.10. Enzyme-Linked Immunosorbent Assay (ELISA)

MPO (Abcam, ab119605) or IL-8 (Absin, abs5100004) ELISA kits were used to evaluate MPO-DNA and IL-8 levels according to the manufacturer's instructions.

### 2.11. Statistical Analysis

The results were shown as means ± SEM. The difference between any two groups was analyzed using a two-tailed unpaired *t*-test. All statistical analyses were carried out using GraphPad Prism 8.0, and *p* < 0.05 was considered statistically significant (⁣^∗^*p* < 0.05, ⁣^∗∗^*p* < 0.01, ⁣^∗∗∗^*p* < 0.001, and ⁣^∗∗∗∗^*p* < 0.0001).

## 3. Results

### 3.1. NETs Formation Is Enhanced in PDAC Tumor Specimens

**Figure 1:**
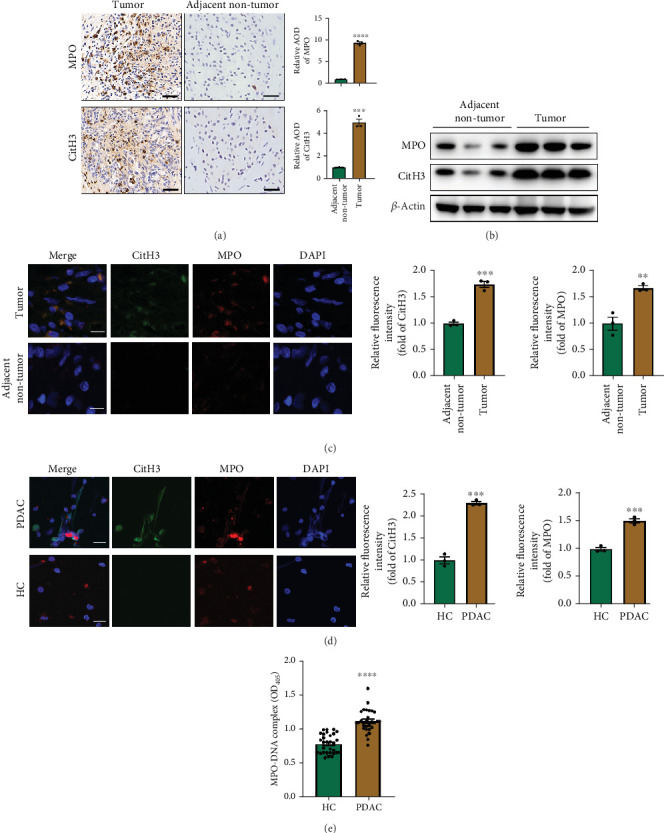
NETs formation is enhanced in PDAC tumor specimens. (a) Representative images of IHC staining of MPO and CitH3 in human PDAC tissue sections and adjacent nontumor tissues. Scale bar: 50 *μ*m. (b) Western blot images of MPO and CitH3 expression from tumor and adjacent nontumor tissues of the same patient. (c) Representative images of IF staining of MPO and CitH3 in tumor and adjacent nontumor tissue sections. Scale bar: 15 *μ*m. (d) Representative IF images of NETs released by neutrophils isolated from PDAC patients and healthy controls (HCs). Scale bar: 15 *μ*m. (e) MPO-DNA levels in serum samples from PDAC patients (*n* = 30) and HCs (*n* = 30). Significant results are presented as ⁣^∗∗^*p* < 0.01, ⁣^∗∗∗^*p* < 0.001, and ⁣^∗∗∗∗^*p* < 0.0001.

NETs have been considered an independent prognostic factor in PDAC [15]. To identify the existence of NETs in PDAC tumors, we performed IHC, IF, and Western blot analyses of NETs indicators, specifically MPO and CitH3, in tissue samples. We found a higher level of NETs in PDAC tissue sections compared with adjacent nontumor tissues (Figures 1(a), 1(b), and 1(c)). Neutrophils were isolated from the blood of PDAC patients or healthy controls (HCs) and subsequently treated with PMA to induce the formation of NETs in vitro. We observed that the neutrophils from PDAC patients exhibited an increased capacity for NETosis compared with those from HC individuals (Figure 1(d)). In addition, the serum level of MPO-DNA, a featured marker for systemic NETs formation, was also found to be significantly elevated in PDAC patients (*n* = 30) compared to HCs (*n* = 30, *p* < 0.0001) (Figure 1(e)). These data indicate an increased formation of NETs in PDAC tumor tissues, and neutrophils derived from PDAC patients possess a heightened potential to form NETs.

### 3.2. NETs Induce EMT to Promote Tumor Invasiveness

**Figure 2:**
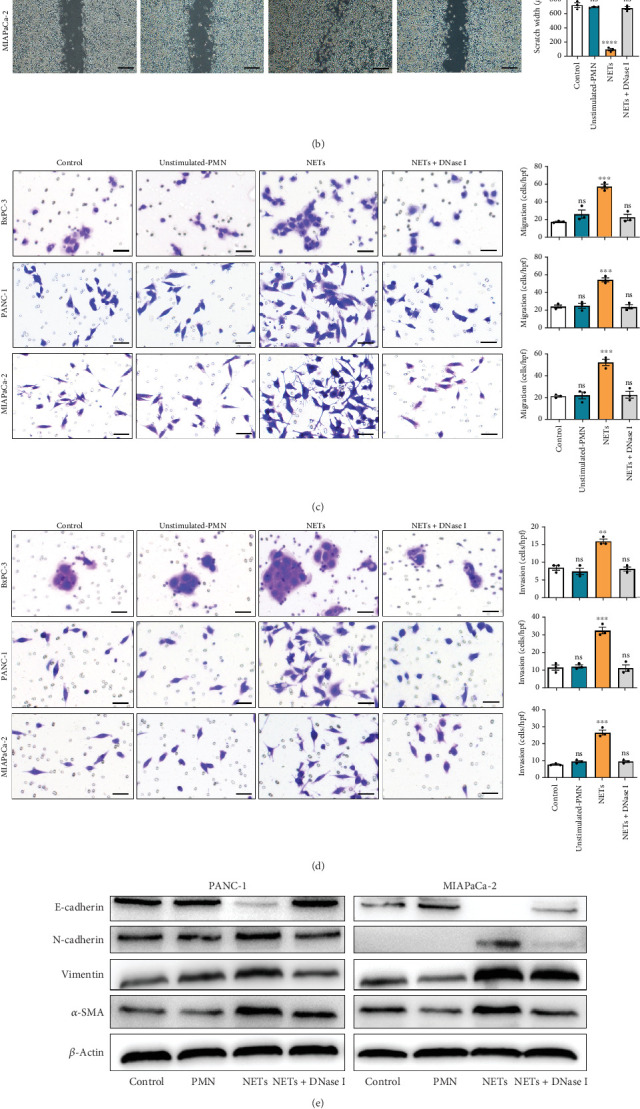
NETs induce EMT to promote tumor invasiveness. (a) Cell proliferation assays of BxPC-3, PANC-1, and MIAPaCa-2 cells treated with unstimulated neutrophils (unstimulated PMN), NETs, or NETs combined with DNase I (NETs + DNase I). (b) Wound healing assays of BxPC-3, PANC-1, and MIAPaCa-2 cells treated with different conditioned mediums. Scale bar: 500 *μ*m. (c, d) Transwell assays of BxPC-3, PANC-1, and MIAPaCa-2 cells treated with different conditioned mediums. Scale bar: 50 *μ*m. (e) Western blot images of E-cadherin, N-cadherin, vimentin, and *α*-SMA expression in PANC-1 and MIAPaCa-2 cells. (f) Representative images of IF staining for PANC-1 cells treated with or without NETs. Scale bar: 10 *μ*m. (g) A heat map of expressions of EMT-related transcription factors in PANC-1 cells. Significant results are presented as ⁣^∗∗^*p* < 0.01, ⁣^∗∗∗^*p* < 0.001, and ⁣^∗∗∗∗^*p* < 0.0001. Nonsignificant results are presented as ns.

Having shown a higher level of NETs in clinical samples, we further investigated whether NETs promoted the proliferation and migration of pancreatic cancer cells. We treated pancreatic cancer cell lines BxPC-3, PANC-1, and MIAPaCa-2 with different mediums, and the results showed that NETs did not accelerate tumor proliferation in all cell lines (Figure 2(a)). Then, we performed experiments to identify the effects of NETs on the migration and invasion capabilities of tumor cells. Wound healing and Transwell assays demonstrated that NETs promoted both migration and invasion abilities of pancreatic cancer cells in vitro, which could be suppressed by the administration of DNase I (1000 U/mL) to degrade NETs (Figures 2(b), 2(c), and 2(d)).

To determine whether NETs influence EMT, we measured levels of several epithelial and mesenchymal markers. Western blot analysis showed that exposure to NETs resulted in the upregulation of mesenchymal markers including N-cadherin, vimentin, and *α*-SMA while downregulating the epithelial marker E-cadherin in PANC-1 and MIAPaCa-2 cells (Figure 2(e)). Additionally, IF analysis corroborated these findings by demonstrating an increased protein level of vimentin alongside a decreased protein level of E-cadherin in PANC-1 cells (Figure 2(f)). What is more, RT-PCR results indicated that NETs led to elevated mRNA levels of Zeb-1, Snail-2, and vimentin in PANC-1 cells (Figure 2(g)). Collectively, these results confirm that NETs can promote invasiveness in pancreatic cancer cells by inducing the EMT program.

### 3.3. NETs-Pretreated Tumor Cells Recruit More Neutrophils

**Figure 3:**
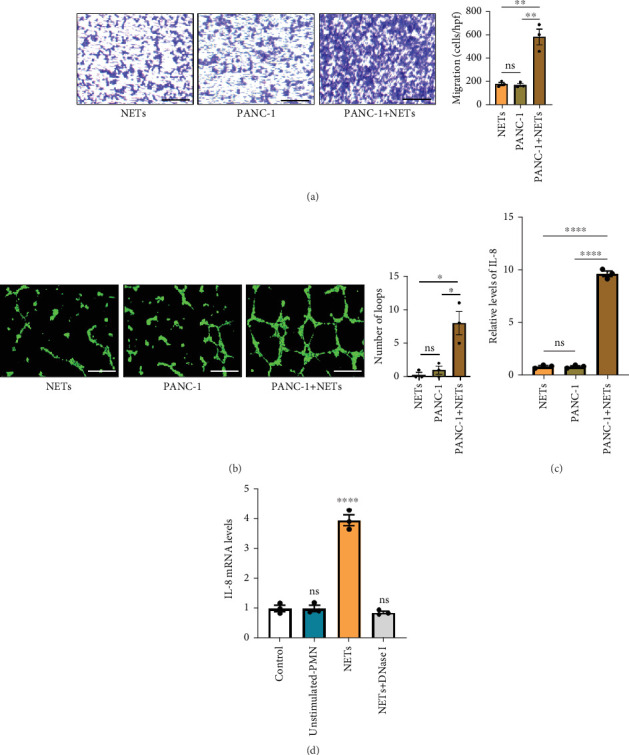
NETs-pretreated tumor cells recruit more neutrophils. (a) Migration of neutrophils treated with conditioned mediums of PANC-1 cells pretreated with NETs (PANC-1 + NETs), PANC-1 cells (PANC-1), and NETs. Scale bar: 100 *μ*m. (b) Angiogenesis of HUVECs in different conditioned mediums. Scale bar: 500 *μ*m. (c) ELISAs of IL-8 levels in different conditioned mediums. (d) The mRNA levels of IL-8 in PANC-1 cells treated with different conditioned mediums. Significant results are presented as ⁣^∗^*p* < 0.05, ⁣^∗∗^*p* < 0.01, and ⁣^∗∗∗∗^*p* < 0.0001. Nonsignificant results are presented as ns.

Our previous study demonstrated that leukocytosis is associated with increased tumor-infiltrating NETs and is an independent prognostic factor for survival in esophageal cancer [16]. Infiltrating leukocytes are regarded as a hallmark of tumors, and the tumor microenvironment (TME) is considered to contribute to tumor initiation, progression, and patient prognosis [17, 18]. Furthermore, cytokines within the TME can recruit more immune cells such as neutrophils. To investigate this further, we evaluated the chemotaxis of neutrophils using a Transwell assay. Our findings revealed that the conditioned medium of PANC-1 cells pretreated with NETs significantly enhanced neutrophil chemotaxis compared with either the medium of PANC-1 cells alone or NETs alone (Figure 3(a)). Meanwhile, human umbilical vein endothelial cells (HUVECs) exposed to the conditioned medium of PANC-1 cells pretreated with NETs exhibited increased angiogenesis (Figure 3(b)). These results suggest that NETs may promote cytokine production in tumor cells.

IL-8 is known to be a classic neutrophil chemokine and has been shown to exert various tumor-promoting functions including angiogenesis [19]. Therefore, the concentration of IL-8 in different conditioned mediums was measured by ELISA, and the results showed that the level of IL-8 in the conditioned medium of PANC-1 cells pretreated with NETs was approximately 10 times higher than the others (Figure 3(c)). RT-PCR analysis also validated the upregulation of IL-8 expression in PANC-1 cells treated with NETs (Figure 3(d)). Taken together, our findings indicate that NETs enable pancreatic cancer cells to secrete IL-8, thereby recruiting more neutrophils into the TME.

### 3.4. NETs Activate the STING Signaling Pathway to Promote IL-8 Secretion

**Figure 4:**
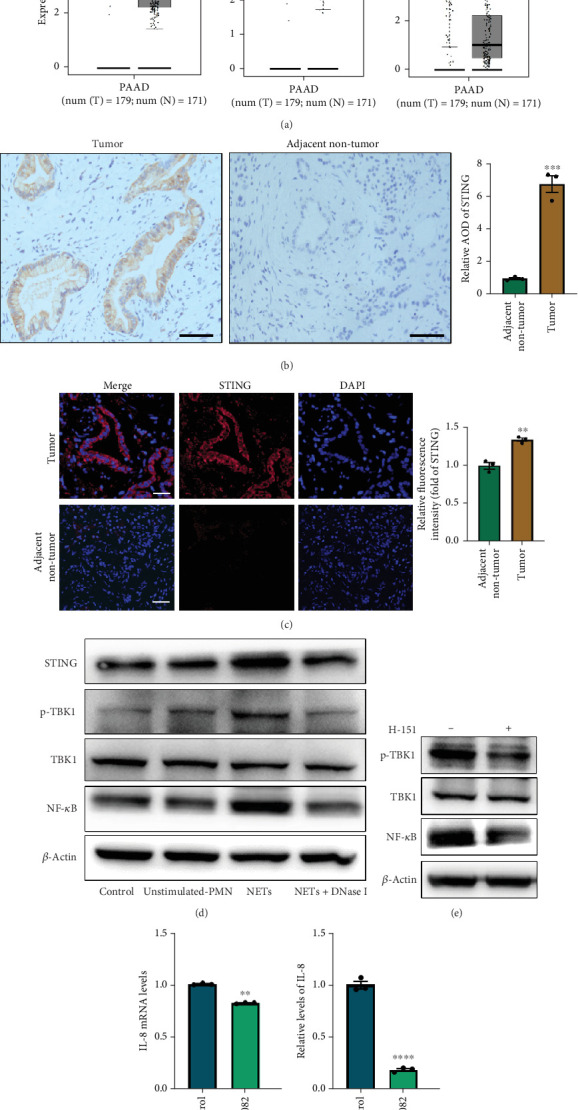
NETs activate the STING signaling pathway to promote IL-8 secretion. (a) STING, NF-*κ*B, and IL-8 expression levels from the GEPIA dataset on PAAD. Representative images of (b) IHC and (c) IF staining of STING in tumor and adjacent nontumor tissue sections. Scale bar: 50 *μ*m. (d) Expression of STING, p-TBK1, TBK1, and NF-*κ*B in PANC-1 cells treated with different conditioned mediums. (e) Expression of p-TBK1, TBK1, and NF-*κ*B in PANC-1 cells with or without H-151 (10 *μ*M, STING inhibitor, MedChemExpress). (f) The mRNA levels of IL-8 in PANC-1 cells treated with or without BAY 11-7082 (25 *μ*M, NF-*κ*B inhibitor, Beyotime). (g) ELISAs of IL-8 levels in the mediums of PANC-1 cells treated with or without BAY 11-7082. Significant results are presented as ⁣^∗^*p* < 0.05, ⁣^∗∗^*p* < 0.01, ⁣^∗∗∗^ *p* < 0.001, and ⁣^∗∗∗∗^ *p* < 0.0001.

There is a strong correlation between persistent inflammation and tumor aggressiveness, with the STING and NF-*κ*B pathways playing important roles in inflammation-driven tumor growth [20, 21]. In addition, NETs could induce activation of the STING pathway, which may subsequently activate NF-*κ*B and thus boost IL-8 production [22]. As expected, data from the Gene Expression Profiling Interactive Analysis (GEPIA) dataset on pancreatic adenocarcinoma (PAAD) showed that patients with pancreatic cancer exhibited increased expression levels of STING, NF-*κ*B, and IL-8 compared with normal controls (Figure 4(a)). IHC and IF analyses confirmed that the expression level of STING in tumor tissues was significantly higher than that in adjacent nontumor tissues (Figures 4(b) and 4(c)). Western blot analysis showed that NETs markedly elevated protein levels of the STING signaling pathway, including STING, p-TBK1, and NF-*κ*B, in PANC-1 cells (Figure 4(d)).

To further explore the involvement of the STING/p-TBK1/NF-*κ*B axis in IL-8 production, we used H-151 (10 *μ*M, STING inhibitor, MedChemExpress) and BAY 11-7082 (25 *μ*M, NF-*κ*B inhibitor, Beyotime) to inhibit STING and NF-*κ*B activation in PANC-1 cells, respectively. We found that the inhibition of the STING pathway markedly reduced protein levels of p-TBK1 and NF-*κ*B (Figure 4(e)). Concurrently, both mRNA and protein levels of IL-8 were decreased upon suppression of the NF-*κ*B pathway (Figures 4(f) and 4(g)). These results collectively demonstrate that NETs induced IL-8 production through the STING/p-TBK1/NF-*κ*B axis in pancreatic cancer cells.

### 3.5. IL-8 Recruits Neutrophils and Mediates NETosis via the MEK/Mitogen-Activated Protein Kinase (MAPK)/Reactive Oxygen Species (ROS) Axis

**Figure 5:**
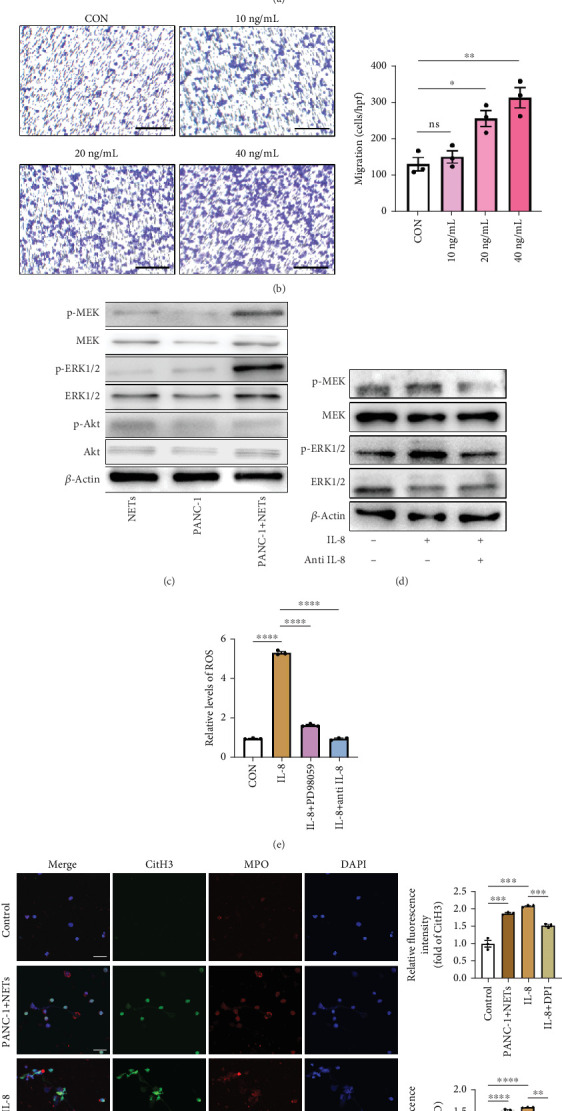
IL-8 recruits neutrophils and mediates NETosis via the MEK/MAPK/ROS axis. (a) Migration of neutrophils treated with conditioned mediums of PANC-1 cells pretreated with NETs with or without an anti-IL-8 antibody. Scale bar: 100 *μ*m. (b) Migration of neutrophils treated with different concentrations of IL-8. Scale bar: 100 *μ*m. (c) Expression of p-MEK, MEK, p-ERK1/2, ERK1/2, p-AKT, and AKT in neutrophils treated with different conditioned mediums. (d) Expression of p-MEK, MEK, p-ERK1/2, and ERK1/2 in neutrophils treated with IL-8 or IL-8 combined with an anti-IL-8 antibody. (e) ROS levels in neutrophils treated with IL-8, IL-8 combined with PD98059 (10 mM, MEK inhibitor, MedChemExpress), or IL-8 combined with an anti-IL-8 antibody. (f) Representative images of IF staining of NETs released from neutrophils treated with conditioned mediums of PANC-1 cells pretreated with NETs (PANC-1 + NETs), IL-8, and IL-8 combined with DPI (10 *μ*M, ROS inhibitor, MedChemExpress). Significant results are presented as ⁣^∗^*p* < 0.05, ⁣^∗∗^*p* < 0.01, ⁣^∗∗∗^*p* < 0.001, and ⁣^∗∗∗∗^*p* < 0.0001. Nonsignificant results are presented as ns.

To clarify whether IL-8 mediated the chemotaxis of neutrophils, Transwell assays were conducted. The addition of the anti-IL-8 antibody to the conditioned medium of PANC-1 cells pretreated with NETs resulted in a reduction of neutrophil chemotactic ability (Figure 5(a)). Furthermore, the chemotaxis of neutrophils elevated with the increase in IL-8 concentration (Figure 5(b)). We treated neutrophils with different conditioned mediums and found that levels of MEK and ERK1/2 phosphorylation increased in neutrophils exposed to the conditioned medium of PANC-1 cells pretreated with NETs, while AKT phosphorylation remained unchanged (Figure 5(c)). Consistent with these results, when IL-8 was added to the medium of neutrophils, enhanced phosphorylation of MEK and ERK1/2 was detected; this effect could be counteracted by the anti-IL-8 antibody (Figure 5(d)).

ROS are known to be a crucial mediator for NETs formation [13, 23], so we further investigated the impact of IL-8 on ROS production in neutrophils. The results indicated that IL-8 led to an increased level of ROS, which could be reversed by a MEK inhibitor (PD98059) or an anti-IL-8 antibody (Figure 5(e)). Both the conditioned medium of PANC-1 cells pretreated with NETs and medium containing IL-8 contributed to NETs formation, while the effect could be suppressed by inhibition of ROS (Figure 5(f)). Together, these findings suggest that IL-8 from the conditioned medium of PANC-1 cells pretreated with NETs promotes NETosis by regulating the MEK/MAPK/ROS axis of neutrophils.

## 4. Discussion

**Figure 6:**
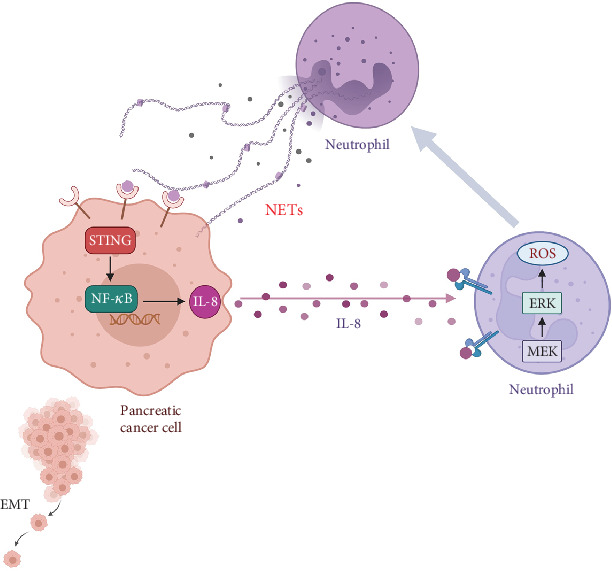
NETs play an important role in the crosstalk between neutrophils and pancreatic cancer cells. A schematic representation illustrating the stimulation of the STING pathway by NETs in pancreatic cancer cells, leading to the secretion of IL-8. IL-8 subsequently enhances neutrophil chemotaxis and promotes NETs formation, ultimately establishing a vicious cycle.

Taken together, our results indicate an enhanced formation of NETs in PDAC tumor samples and a higher potential of neutrophils derived from PDAC patients to form NETs. NETs could promote migration and invasion of pancreatic cancer cells as well as EMT. In addition, NETs stimulated tumor cells to secrete IL-8, which in turn increased the chemotaxis of neutrophils, thereby establishing a vicious cycle (Figure 6). Therefore, our results suggest that targeting NETs could be a viable strategy for mitigating tumor progression.

Together with macrophages, neutrophils are the most abundant type of immune cells and can be activated by various stimuli within the TME of PDAC, facilitating poor prognosis [24, 25]. There is strong evidence supporting the involvement of neutrophils in every step of the metastatic cascade, including invasion and intravasation, establishment of premetastatic niches, extravasation, and final recurrence [26, 27]. The cellular interactions between neutrophils and tumor cells as well as with other immune cells enhance metastasis in several ways [10, 28]. However, the specific role of neutrophils in PDAC metastasis remains to be elucidated. In this study, we demonstrated that neutrophils promote tumor invasiveness via NETosis, which could be abrogated through DNase I digestion. In addition to neutrophils, future studies are necessary to observe the status of infiltration of lymphocytes and macrophages in tumor tissues.

NETs are web-like structures containing decondensed chromatin filaments coated with histones. They are released by activated neutrophils and possess the ability to trap and kill pathogens, along with other cells [11, 29]. Beyond that, NETs have been linked to other malignancies as a double-edged sword, promoting tumor growth, metastasis, angiogenesis, cancer-associated thrombosis, and organ dysfunction through regulating the TME and antitumor immune responses [30–32]. NETs in cancer patients can be induced by tumor-derived cytokines and further exacerbated by surgical interventions and infections.

Numerous studies focus on the mechanisms underlying the action of NETs in cancer, including pancreatic cancer. Related research has shown elevated levels of NETs in mice bearing human pancreatic tumors, which enhanced venous thrombosis. Notably, depletion of neutrophils or DNase I treatment reduced venous thrombosis [33]. Other investigations have indicated that NETs act as promoters of cancer metastasis and recurrence by awakening dormant cancer cells [34]. Therefore, it is plausible to consider NETs as an independent prognostic factor and a promising therapeutic target for pancreatic cancer. In the present study, we mainly examined the metastatic behavior of pancreatic cancer cells and confirmed that NETs could promote both migration and invasion while simultaneously inducing EMT. Although NETs may promote metastasis through distinct mechanisms, EMT might represent an initial step in tumor dissemination [35]. Here, the expression of EMT-related proteins, E-cadherin, was downregulated, while N-cadherin, vimentin, and *α*-SMA were upregulated in pancreatic cancer cells treated with NETs. However, further investigation is warranted to elucidate the precise mechanism by which NETs facilitate EMT.

It is estimated that at least 20% of cancers are directly attributable to various chronic inflammatory diseases [36]. Pancreatitis or chronic inflammation has been recognized as a risk factor for pancreatic cancer [37–39]. Increasing evidence indicates that the inflammatory state within the TME, including inflammatory cells and molecules, aids in tumor metastasis [40]. NETs may serve as critical components of the TME, influencing its immune modulation. As our results show, NETs triggered pancreatic cancer cells to produce IL-8 by activating the STING pathway, which in turn recruited more neutrophils and promoted NETosis. Tumor-derived cytokines, including IL-8, have been shown to correlate with tumor-induced NETs formation and tumor aggressiveness [41]. This implies that neutrophils infiltrating tumors may be reprogrammed by these tumor-derived factors. Mechanistically, our findings demonstrated that IL-8 activated neutrophils toward forming NETs via the MEK/MAPK/ROS signaling pathway. In conclusion, it is conceivable that NETs are intricately linked to inflammation and cancer progression. Furthermore, the proinflammatory milieu of the TME can recruit neutrophils and enhance NETs formation [42]. Thus, in this vicious cycle, pinpointing a singular initiating factor proves challenging. Nevertheless, substantial evidence supports the notion that NETs play a pivotal role in this complex interplay.

All in all, pancreatic cancer poses a threat to human health due to its high mortality rate. Therefore, our objective is to enhance the understanding of the underlying dysregulation mechanisms. In this study, we first demonstrated that NETs activate the STING pathway of pancreatic cancer cells to produce IL-8, which recruits additional neutrophils into the TME. Ultimately, this positive feedback loop between neutrophils and pancreatic cancer cells accelerates tumor progression. Consequently, targeting NETs presents a potential therapeutic strategy for treating pancreatic cancer; however, it remains challenging to identify a therapeutic window where the bactericidal function of NETs is not weakened.

## Data Availability

All relevant data of this study are included in this article.
